# Editorial: Non-invasive methods in drug metabolism and transport: insights from biological samples to oral administration

**DOI:** 10.3389/fphar.2025.1735743

**Published:** 2025-11-12

**Authors:** Karol Wróblewski, Yi Zheng, Xuyi Yue

**Affiliations:** 1 Laboratory of Commercial and Non-commercial Clinical Trials, Faculty of Medicine, University of Rzeszów, Rzeszów, Poland; 2 Qilu Pharmaceutical Co. Ltd., Shanghai, China; 3 Translational Neuroimaging Laboratory, Department of Radiology, Nemours Children’s Health, Wilmington, DE, United States

**Keywords:** drug metabolism, transport of drugs, biological samples, oral administration, drug determination

The study of the metabolism and transport of drugs, endogenous compounds, and environmental chemicals is crucial for understanding pharmacokinetics, optimizing therapeutic outcomes, and reducing the risk of adverse effects. A more comprehensive understanding of drug metabolism and transport within the body may be possible by combining non-invasive methods with oral administration research. Non-invasive sampling methods include urinalysis, saliva analysis, hair analysis, and breath testing. The qualification or quantification of drugs, their metabolites, and other compounds in these samples can be performed using various analytical techniques such as liquid chromatography (LC), gas chromatography (GC), capillary electrophoresis (CE), and mass spectrometry (MS) (Alanazi, 2025; Hancu et al., 2021; Hadland and Levy, 2016; Moura et al., 2023; Wróblewski et al., 2023). In addition to non-invasive sampling techniques, oral medication administration offers both opportunities and challenges for advancing our understanding of drug metabolism and transport. Oral drug administration is the preferred route for drug delivery because it is convenient and widely accepted by patients (Kim and De Jesus, 2025). However, various anatomical, biochemical, and physiological factors affecting oral drug delivery must be taken into account (Lou et al., 2023). To ensure efficacy and safety, thorough studies of bioavailability, absorption, distribution, and first-pass metabolism are necessary.

This Research Topic includes five manuscripts that cover interdisciplinary studies on non-invasive methods in drug metabolism and transport. In the first study, Jerszyńska and Szultka-Młyńska conducted an electrochemical simulation of psychotropic drug metabolism and compared it to *in vivo* processes using liquid chromatography-tandem mass spectrometry (LC-MS/MS). Electrochemistry (EC) and liver microsome tests were utilized to produce transformation products (TPs), which are byproducts of the breakdown of psychiatric drugs, to examine the possible TPs of target drugs. These TPs were then further characterized using LC-MS/MS. By examining biological samples (human plasma) from patients, the outcomes of EC-(LC)-MS and liver microsome assays were found to be comparable to those of traditional *in vivo* investigations. Data from electrochemical oxidation, which predicted some of the possible metabolites present in the human liver microsomes, concurred with data from *in vivo* test results. The oxidative metabolism of certain psychiatric medications can be accurately simulated using EC–(LC)-MS. Reactive TPs can be directly identified with EC-(LC)-MS, which eliminates the necessity for animal testing.

In a mouse model of dyslipidemia, metabolomics and network pharmacology revealed the hypolipidemic mechanisms of ferulic acid (FA) (Zeng et al.). In mice with hyperlipidemia induced by the triton WR-1339, the lipid-lowering mechanism of FA was investigated by integrating network pharmacology and metabolomics. Additionally, 31 distinct metabolites, including those involved in lipid and amino acid metabolism, were examined in relation to FA’s effects on hyperlipidemia. Following oral administration of FA, its primary function in resolving metabolic problems and preserving the dynamic equilibrium of metabolites was investigated. The results show that FA is linked to lipid metabolism, including the metabolism of ether lipids, arachidonic acid, and linoleic acid. Using Kyoto Encyclopedia of Genes and Genomes (KEGG) analysis, the main signaling pathways of FA against hyperlipidemia were identified as lipid and atherosclerosis signaling pathways. Conjoint investigation revealed that six biomarkers and 18 key targets were linked to FA’s ability to prevent hyperlipidemia. FA showed a strong binding affinity for these key targets, as indicated by the results of molecular docking.

Zhao et al. investigated the effects of acute hypobaric hypoxia (HH) on the anti-fatigue properties of pitolisant and explored the underlying mechanisms. In sleep-deprived mice under HH conditions, pitolisant significantly improved learning, memory, cognition, and motor function (P < 0.05). Under HH circumstances, however, pharmacokinetic studies showed decreased pitolisant levels in the brain. By suppressing the expression of P-glycoprotein and organic cation transporter 1, HH reduced the anti-fatigue benefits of pitolisant. These findings demonstrate its usefulness in high-altitude areas where improved cognitive function is required.

For patients with cystic fibrosis (CF), excipient-free tobramycin dry powder inhalers (DPIs) may be a promising treatment option for *Pseudomonas* aeruginosa-induced lung infections (Cheng et al.). With a minimum inhibitory concentration (MIC) of 0.5 μg/mL, the inhalable powder demonstrated exceptional safety performance in both *in vivo* and *in vitro* safety and activity experiments at the animal and cellular levels. Inhalation of excipient-free tobramycin inhalable powder had a better effect in the infected mouse model than intravenous injection, likely due to its amorphous state. The results of this research show that non-invasive routes of administration (in this case, inhalation) can improve local concentrations, reduce systemic exposure, bypass first-pass metabolism, and ultimately improve treatment efficacy.

The review by Idasiak-Piechocka et al. highlighted the relevance of hypoalbuminemia in therapeutic efficacy and safety by examining its effects on the pharmacokinetics of several medications, including antibiotics, immunosuppressants, antifungals, and anticonvulsants. The review describes the changes in pharmacokinetic parameters observed in hypoalbuminemia and can serve as a guide for medical professionals. According to the study, patients with hypoalbuminemia require customized dosage schedules based on therapeutic drug monitoring (TDM) to maximize medication therapy and prevent toxic or subtherapeutic drug levels. Drug dosage must be adjusted, particularly in diseases where hypoalbuminemia is common, such as cirrhosis, sepsis, or nephrotic syndrome.

The articles in this Research Topic illustrate significant progress in the field of non-invasive methods in metabolism and transport. By combining advanced analytical techniques (e.g., chromatography, electrochemistry, and mass spectrometry), innovative drug manufacturing and delivery strategies (e.g., inhalation powders), and network pharmacology, in addition to highlighting physiological and pharmacological modulators, researchers are moving toward more ethical, patient-friendly, and clinically applicable paradigms.
